# The Medical Remembrancer, or Book of Emergencies

**Published:** 1845-12

**Authors:** 


*The Medical Remembrancer or Book of Emergencies.*—By EDWARD B. S. SHAW, M. R. C. S., & L. A. S., &c. &c.—Revised and improved by an American Physician. New York: Samuel S. & William Wood, 1845. pp. 112, 24mo. (From the publishers.)

This little volume professes to be a practical guide to young practitioners in cases of emergency. It presumes a knowledge of the diagnostic symptoms of the emergent cases treated of in its pages. The work contains four chapters, in which the arrangement of the different subjects is alphabetical, for the greater convenience of reference. Chapter 1 is on the immediate treatment of poisons. Chapter 2, immediate treatment of accidents. Chapter 3, on the minor operations. Chapter 4, on chemical analysis and the tests for the principal poisons. The convenience and usefulness of this little manual is undoubted, *if* it is used *only* as a *remembrancer*, and, as such, we recommend it with pleasure. We must however express our decided objection to the habitual use of small and condensed works, giving but a partial view of the subjects discussed, and our doubts of the propriety of the increase in the number already in circulation. The temptation to use them, in place of larger and more complete works, to the student preparing for his examination, and to the practitioner busily occupied in the arduous duties of his profession is too great to be easily avoided, and the necessary result is that both become superficial, and thereafter are indisposed to more profound study.

The work before us is one of the best of its class, and as far as it professes to go, appears to be accurate and reliable.—ED.

